# Isolation, characterization, and LC MS/MS determination of anti-obesity components from pine needles of *Cedrus deodara* (Roxb.) G. Don

**DOI:** 10.3389/fnut.2024.1448908

**Published:** 2024-08-01

**Authors:** Xin Wang, Bin Li, Dongyan Liu, Yuer Guo, Jiaxu Zhang, Wanyu Li, Tengteng Peng, Quhuan Ma, Xiaofeng Shi

**Affiliations:** ^1^College of Pharmacy, Gansu University of Traditional Chinese Medicine, Lanzhou, China; ^2^Institute of Pharmaceutical research, Gansu Academy of Medical Sciences, Lanzhou, China; ^3^College of Biomedicine, Lanzhou University of Technology, Lanzhou, China; ^4^College of Life Science, Northwest Normal University, Lanzhou, China

**Keywords:** *Cedrus deodara* (Roxb.) G. Don, needle leaves, anti-obesity ingredients, phenolic acids, UPLC-QQQ-MS/MS

## Abstract

**Objective:**

This study aimed to isolate and analyze the components in cedar pine needles (needle leaves of *Cedrus deodara* (Roxb.) G. Don) that exhibit anti-obesity effects, as determined through animal experiments.

**Methods:**

The extract of cedar pine needles was separated into four fractions of different polarities using a macroporous resin column. The fraction that retained anti-obesity activity was evaluated based on the results of animal experiments. Monomeric compounds were structurally characterized and isolated from the active fraction using a preparative liquid chromatography system. Combined with subsequent glucose gel chromatographic separation. The content of the separated components was determined using ultrahigh performance liquid chromatography coupled with triple quadrupole mass spectrometry (UPLC-QQQ-MS/MS).

**Results:**

The water-washed fraction retained anti-obesity activity of the cedar pine needles more effectively. A total of 16 compounds were separated from this fraction, and the contents of 14 of these compounds were determined to be present in cedar pine needles.

**Conclusion:**

Nine components, namely p-hydroxy benzyl alcohol, chlorogenic acid, vanillic acid, syringic acid, P-coumaric acid, sinapic acid, benzoic acid, phenylacetic acid, salicylic acid, were characterized and determined for the first time in cedar pine needles. The components with anti-obesity activity in the pine needles of *Cedrus* are mainly derived from phenolic acids.

## 1 Introduction

*Cedrus deodara* (Roxb.) G. Don is an evergreen tall arbor with branches and leaves that are spreading, slightly slanting, or slightly drooping. Its leaves are blue-green and needle-shaped, which are spirally arranged on long branches and clustered on short branches. The cones are upright, green before maturity and reddish-brown when mature. It is monoecious, with flowers solitary on the top of branches, and its flowering period is from October to November. *Cedrus deodara* is named for its cold tolerance and is native to regions approximately 2,000 m above sea level in Afghanistan and the Himalayas. It is now widely found in many countries in the Northern Hemisphere as a greening tree species.

*Cedrus deodara* was first described by Trew (1, 2). Pine needles of cedar have been regarded as an important medicinal and edible homologous natural product by ancient people throughout human history. As a common resource, pine needles, particularly those from cedar pines, are widely used in many countries and regions around the world (3). In traditional Chinese medicine, its efficacy is described as “dispelling rheumatism, regulating visceral Yin and Yang, relieving hunger, and prolonging lifespan.” Modern pharmacological studies have shown that pine needles have various properties, including anti-cancer (4, 5), anti-fungal (6), anti-arthritic (7), anti-allergic (8, 9), spasmolytic and anti-inflammatory (10), and anti-oxidant (11, 12), and is used in the management of animal diseases (13–15).

**SCHEME 1:**
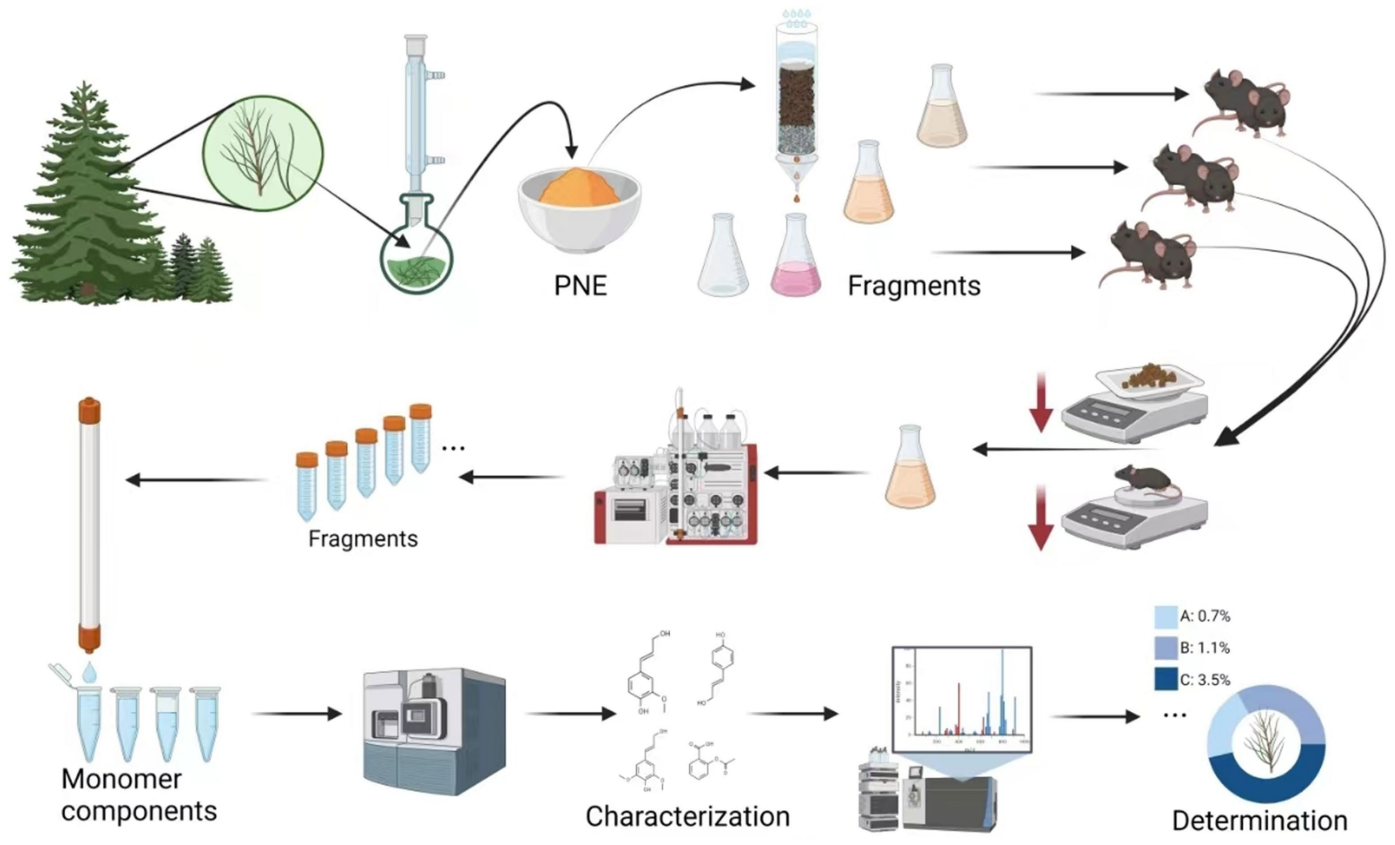
Schematic illustration of the isolation, characterization, and determination of anti-obesity components from cedar pine needles.

However, there is insufficient scientific evidence regarding the regulation of metabolism by cedar pine needles. In our preliminary experiments, we found that administering cedar pine needle powder to mice via gavage at a dose of 0.6 g/kg per day could reduce the amount of food consumed and body weight in normal mice. This function was confirmed through a comparative experiment with nutritional supplements, indicating that the observed effects are not related to the nutrient components contained in cedar pine needles. Currently, there are insufficient reports on the nutrient metabolic activity of pine needles (16, 17). In this study, we traced and characterized the potential components in cedar pine needles that can reduce obesity (Scheme 1).

## 2 Materials and methods

### 2.1 Reagents

The reagents used in the experiment include Mouse GLP-1 ELISA Kit, lot number: mL003047V; Mouse leptin (LEP) ELISA Kit, lot number: mL002969V; Mouse Ghrelin ELISA Kit, lot number: mL059461V (Enzyme-linked Biotechnology Co., Ltd., Shanghai, China), rss© 2008–2022; and Sephadex LH-20 (GE Healthcare, United States, Lot:10233922).

The other materials used in the experiment are as follows: gallic acid (C17D10C105977, ≥98% w/w), p-hydroxybenzyl alcohol (MUST-22111517, 99.88% w/w), protocatechuic acid (MUST-23083012, 99.99% w/w), P-hydroxybenzoic acid (MUST-23070408, 99.97% w/w), chlorogenic acid (MUST-22111711, 99.82% w/w), vanillic acid (MUST-23012113, 99.62% w/w), caffeic acid (MUST-23061118, 99.82% w/w), syringic acid (MUST-23033115, 99.81% w/w), P-coumaric acid (MUST-23033113, 99.99% w/w), sinapic acid (MUST-22110218, 99.94%), benzoic acid (106419-202003, 99.90%), phenylacetic acid (MUST-23092508, 98.56% w/w), salicylic acid (MUST-23040202, 99.91% w/w), trans-Cinnamic acid (MUST-23071910, 99.99% w/w); acetonitrile (gradient grade for liquidchromatography, Lichrosolv. JA087530, Merck KGaA.), methanol (gradient grade for liquidchromatography, Supelco. L1084107 021, Merck KGaA.), fomic acid (gradient grade for liquidchromatography, YRJGH-WS F0513 TCI Shanghai), pure water (Milipore), and a 0.22-μm microporous filter.

#### 2.1.1 Experimental animals

In this study, 16-week-old adult male mice weighing 21.08 ± 1.47 g were used. The experiments were conducted in a laboratory environment at 25 ± 2°C with the light:dark ratio of 14:10 h. All mice had *ad libitum* access to food and water. Animal-related experiments in this study were approved by the Animal Ethics Committee of the Gansu University of Traditional Chinese Medicine. The mouse growth and reproduction diets were provided by Beijing Cooperative Feed Co., Ltd. (moisture ≤10%, crude protein ≥20%, crude fat ≥4%, crude fiber ≤4%, lysine ≥1.32%, methionine and cysteine ≥0.78%, and total ash ≤8%) (SPF License No.: SCXK (Liaoning) 2022-0001, Lot No. 88001234).

### 2.2 Instruments and software

The instruments and software used are as follows: CPA225D Electronic Balance (Hundred-thousandth Precision, Sartorius Co., Germany); Agilent 1260 Infinity II DAD detector, Agilent 1260 FC-PS preparative scale fraction collector, Binnasi-BS C_18_ collum Biosepur, China; Agilent 1290 UHPLC-Agilent6460-Triple Quad Mass Spectrometry (Agilent Technologies, United States); HK3310LHC Ultrasonic Bath (Supmile Co., China); IKA RV 10 auto Rotary Evaporator (IKA, Germany).

### 2.3 Plant materials

The pine needles used in the experiment were collected from Lanzhou City (Sample 1, S1) and Tianshui City (Sample 2, S2) in Gansu Province. Dr. Xiaofeng Shi of the Institute of Pharmaceutical Science, Gansu Academy of Medical Sciences, identified them as the needles of the *Cedrus deodara* (Roxb.) G. Don.

### 2.4 Extraction and isolation of cedar pine needles

We collected fresh cedar pine needles, washed them with clean water, and allowed them to air dry naturally. The dried cedar pine needles were cut into pieces smaller than 2 cm in length. We took 5 kg of this sample, soaked it in 60 L of 40% ethanol solution for 2 h, and then boiled it for 1 h. Subsequently, we separated the extract, added 50 L of 40% ethanol solution, and boiled it again for 1 h. Then, we combined the two extracts, concentrated them under reduced pressure until dry, powdered them (PNE), weighed them, and calculated the yield (18). We then took 0.5 kg of PNE, suspended it in purified water, added it to a pre-prepared HPD7-22 macroporous resin column (Ø15 cm × 100 cm), and eluted it with pure water (6 BV), 5% ethanol (6 BV), 30% ethanol (4 BV), and 70% ethanol (4 BV). Afterward, we collected the eluates and concentrated them under reduced pressure (using water at 65°C, −90 bar) to 500 mL.

### 2.5 Selection of the active fraction

After 7 days of acclimatization feeding, 48 male C57BL/6j mice with similar body weights and in good condition were randomly divided into six groups: the vehicle group, cedar pine needle powder-pure water suspension (SZ), water-washed fraction (WF), 5% fraction (F), 30% F, and 70% F groups, with eight mice in each group. Cedar pine needle powder was ground and passed through a 100-mesh sieve and then suspended in 20 mL of pure water at a concentration of 5 g. The mice were fed once a day at 16:00, and the above groups were given pure water (vehicle), cedar pine needle powder-pure water suspension (SZ, 10 g → 40 mL) at 0.01 mL/g., WF, 5% F, 30% F, 70% F, all of which were 20 times diluted to match the concentration of SZ. Each group of mice was administered orally twice a day at a dose of 20 mL/Kg. The mice were continuously administered for 10 weeks, and their weight, daily food intake, body fat, and biochemical indicators were observed to determine the activity of each fraction.

Blood was collected by cardiac puncture and decapitation after the animals were fully anesthetized with ketamine and xylazine (87.0 + 13.0 mg/kg). Dissection and observation of fatty tissue were performed. Serum was centrifuged at 400 r/min after resting for half an hour, and the serum levels of GLP-1, LEP, and ghrelin were determined using an enzyme-linked immunosorbent assay (ELISA). All kits were used for a double antibody one-step sandwich ELISA, and the absorbance (OD) was measured at a wavelength of 450 nm with an enzyme-labeled instrument to calculate the sample concentration. The daily food intake and body weight of the mice were continuously recorded.

### 2.6 Purification of components

The water-washed fraction (WF) was diluted 10 times and filtered through a 0.45-μm microfiltration membrane. It was then separated using an Agilent 1260 preparative HPLC equipped with a 1260 FC-PS preparative scale fraction collector. The mobile phase consisted of solution A (methanol) and solution B (water), using gradient elution as follows: 0 ~ 15 min, 5%A ~ 23%A; 15.01 ~ 18 min, 23%A ~ 100%A; 18.01 ~ 26 min, 5%A ~ 5%A. The flow rate was 5.0 mL·min^−1^, and 14 fractions were collected. After drying and concentrating each fraction, it was purified using Sephadex LH-20 glucan gel as the stationary phase (Ø1 cm × 100 cm), eluted with 50% methanol, and subjected to repeated purification. The purified components were observed under a UV lamp (254 nm, 365 nm) and then recrystallized to obtain the monomeric compounds.

### 2.7 Characterization of isolated components

The separated compounds were analyzed using mass spectrometry, nuclear magnetic resonance (NMR) hydrogen spectrum, and NMR carbon spectrum techniques. The molecular ion charge-to-mass ratio, fragmentation patterns, fragment ion charge-to-mass ratio, hydrogen atomic chemical environment and chemical shift, and carbon atomic chemical environment and chemical shift were interpreted from the spectral data. The structure of the monomeric compounds was characterized by referring to previous literature reports.

### 2.8 Preparation of a standard solution

We accurately weighed 17.7 mg of cinnamic acid standard and placed it in a 50-mL volumetric flask. We then added 30 mL of 50% methanol and waited for dissolution. Then, we added 50% methanol up to the mark, shake well, and filter through a 0.22-μm filter. We took 0.5 mL of the filtrate and placed it in a 50 mL volumetric falsk, (Confusing description).

We accurately weighed the following components as follows: 2.12 mg of gallic acid; 5.37 mg of p-hydroxybenzyl alcohol; 3.02 mg of protocatechuic acid; 11.48 mg of p-hydroxybenzoic acid; 10.60 mg of chlorogenic acid; 3.43 mg of vanillic acid; 3.49 mg of caffeic acid; 3.16 mg of syringic acid; 5.30 mg of p-coumaric acid; 10.12 mg of benzoic acid; 10.90 mg of phenylacetic acid; and 1.88 mg of salicylic acid.

We placed all these compounds in the 50-mL volumetric flask Pre-add 0.5 mL of cinnamic acid. We added 40 mL of 50% methanol solution and ultrasonicated it for 5 min. We allowed the solution to stand at room temperature (19, 20). We continued to add 50% methanol to the mark, shake well, and filter through a 0.22-μm filter for further analysis.

### 2.9 Preparation of the sample solution

All crude cedar pine needle samples were powdered to a homogeneous size using an electrical mill, sieved through a 100-mesh sieve, and dried at 40°C until they reached constant weight. We accurately weighed 0.5 g of this cedar pine needle sample and placed it in a conical flask with a stopper. We then added 50 mL of 50% methanol, precisely weighed it, and ultrasonically extracted it for 15 min (the output power was set at 40 kHz, without heating). We removed the flask and allowed it to stand at room temperature (19). We precisely weighed the solution again and used 50% methanol to compensate for any loss in weight. We shook the mixture well, filtered it through a 0.22-μm filter, precisely measured 2 mL of the filtrate, and placed it in a 10-mL volumetric flask. Afterward, we added 50% methanol up to the mark, shook it well, and set it aside for later use (S1 and S2 were prepared).

### 2.10 UPLC method development

The phenolic acid in the WF was analyzed using an Agilent 1,290 Series HPLC system (Agilent Technologies, USA). The UPLC pumps, autosampler, and column oven system were monitored and controlled using Agilent MassHunter workstation data acquisition software (Agilent Technologies) (21). The column used was a Waters ACQUITY UPLC BEH HILIC (1.7 μm, 2.1 mm × 100 mm). The auto sampler temperature was set to 4°C, and the oven temperature was maintained at 35°C. The mobile phase consisted of 0.1% formic acid-acetonitrile (A) and 0.1% formic acid-water solution (B). Gradient elution was conducted as follows: 0–8 min, 5%A–13%A; 8–12 min, 13%A-23%A; 12–21 min, 23%A-35%A; and 21–26 min, 35%A–100%A. The flow rate was 0.2 mL·min^−1^, and the injection volume was 2 μL.

### 2.11 MS method development

A QQQ-MS/MS system equipped with an Agilent Jet Stream Source (AJS) electrospray ionization (ESI) source was used, operating in both positive and negative ion scanning modes and multiple reaction monitoring (MRM) mode. The instrument settings were as follows: ionization voltage, 5,500 V (positive) and − 4,500 V (negative) (22). The ion source temperature was set to 450°C. The spray gas was nitrogen (N_2_) at a pressure of 55 psi, the auxiliary heating gas was also N_2_ at a pressure of 55 psi, and the curtain gas was N_2_ at a pressure of 30 psi (19). Data acquisition and analysis were conducted using Agilent MassHunter workstation data acquisition software (Agilent Technologies). The mass spectrometry analysis parameters are listed in Table 1.

**Table 1:** Parameters for mass spectrometry analysis.

Component	RT (min)	Parent ion (*m/z*)	Fragmentor	Daughter ion (*m/z*)	Collision energy (eV)	Polarity
Gallic acid	2.548	169.00	90	^**※**^125.0[-C_6_H_5_O_3_]^−^	15	Negative
				79.1[-C_5_H_3_O]^−^	25	
p-Hydroxybenzyl alcohol	3.365	122.9	80	^**※**^105.1[-C_7_H_5_O]^+^	10	Positive
				77.2[-C_6_H_5_]^+^	15	
Protocatechuic acid	4.896	153.00	90	^**※**^109.1[-C_6_H_5_O_2_]^−^	15	Negative
				91.1[-C_6_H_3_O]^−^	25	
p-Hydroxybenzoic acid	7.496	137.00	80	^**※**^93.1[-C_6_H_5_O]^−^	15	Negative
				65.2[-C_5_H_5_]^−^	30	
Chlorogenic acid	8.217	353.15	140	^**※**^190.9[-C_7_H_10_O_6_]^−^	15	Negative
				85.2[-C_4_H_5_O_2_]^−^	46	
Vanillic acid	9.043	166.90	80	^**※**^151.8[-C_7_H_4_O_4_]^−^	10	Negative
				108.2[-C_6_H_4_O_2_]^−^	16	
Caffeic acid	9.496	179.0	80	^**※**^135.1[-C_8_H_6_O_2_]^−^	15	Negative
				89.2[-C_4_H_9_O_2_]^−^	35	
Syringic acid	9.800	197.0	80	^**※**^122.9[-C_7_H_7_O_2_]^−^	22	Negative
				167.0[-C_8_H_7_O_4_]^−^	18	
p-Coumaric acid	12.713	163.0	80	^**※**^119.2[-C_8_H_6_O]^−^	24	Negative
				93.2[-C_6_H_5_O]^−^	38	
Sinapic acid	14.226	223.0	80	^**※**^208.1[-C_10_H_9_O_5_]^−^	15	Negative
				193.0[-C_10_H_9_O_4_]^−^	22	
Benzoicacid	15.426	122.12	65	^**※**^82.0[-C_5_H_6_O]^−^	15	Negative
				77.2[-C_6_H_5_]^−^	26	
Phenylacetic acid	16.652	135.0	80	^**※**^91.3[-C_7_H_7_]^+^	15	Positive
				65.3[-C_5_H_5_]^+^	40	
Salicylic acid	17.491	137.0	80	^**※**^93.1[-C_6_H_5_O]^−^	14	Negative
				65.2[-C_5_H_5_]^−^	30	
Cinnamic acid	21.261	147.0	80	^**※**^103.2[-C_8_H_7_]^−^	10	Negative
				77.2[-C_6_H_5_]^−^	20	

“^**※**^**”** for quantitative ions.

### 2.12 Statistical analyses

The data from animal experiments were expressed as the average ± standard deviation of at least three individual measurements and were analyzed using descriptive statistics and a one-way analysis of variance (ANOVA) with Statistical Package for the Social Sciences (SPSS) software, version 22.0 (IBM, United States).

### 2.13 Method validation

#### 2.13.1 Specificity

To assess specificity, a blank (50% v/v methanol, n = 2), a mixture of standard solution (50% v/v methanol, n = 2), and a sample solution (50% v/v methanol, n = 2) were tested to check for any interference from the blank or sample.

#### 2.13.2 LOQ and LOD

To determine the limits of quantification (LOQ) and limits of detection (LOD), standard solutions were diluted to various concentrations for a sensitivity test. An increasing concentration of standard solution was injected individually until a signal-to-noise ratio (S/N) of 10.0 and 3.0 was obtained for the determination of LOQ and LOD, respectively.

#### 2.13.3 Repeatability of the experiment

Six parts of pine needle S1 samples were carefully weighed at 0.50 g each and placed in conical flasks with plugs. Following the sample preparation method, the samples were prepared and diluted accordingly, each sample was test three times, and the AUC of each component were averaged. The relative standard deviations (RSDs) of each content in the six samples were then calculated.

#### 2.13.4 Linearity and range

We precisely pipetted 20.0 mL, 10.0 mL, 5.0 mL, 2.5 mL, and 1.0 mL of gallic acid, p-hydroxybenzyl alcohol, protocatechuic acid, p-hydroxybenzoic acid, chlorogenic acid, vanilla acid, caffeic acid, syringic acid, p-coumaric acid, sinapic acid, benzoic acid, phenylacetic acid, salicylic acid, and trans-cinnamic acid reserve solutions into 100 mL volumetric flasks. We then diluted each with 50% methanol, shook them well, and let them stand. We analyzed the standard and diluted solutions mentioned above, determining a linear equation and correlation coefficient (R2) using SPSS 22.0 software.

#### 2.13.5 Accuracy

The accuracy of the developed method was evaluated through an analyte recovery test at three concentration levels. A known amount of nine samples of S1 were precisely weighed, 0.2 g each. Each group consisted of three samples, each spiked with the standard substances at low, medium, and high concentrations, corresponding to 0.8, 1.0, and 1.2 times the content, respectively. The samples were prepared and diluted according to the “2.9 sample solution” preparation method.

## 3 Results and discussion

### 3.1 Extraction and isolation of cedar pine needles

A reddish-brown powder of total pine needle polyphenols was obtained by extraction, yielding 19.7% of the dry pine needle mass. Four fractions were obtained by elution, marked as WF, 5% F, 30% F, and 70% F, and frozen for later use.

### 3.2 Selection of active fraction

**Figure 1:**
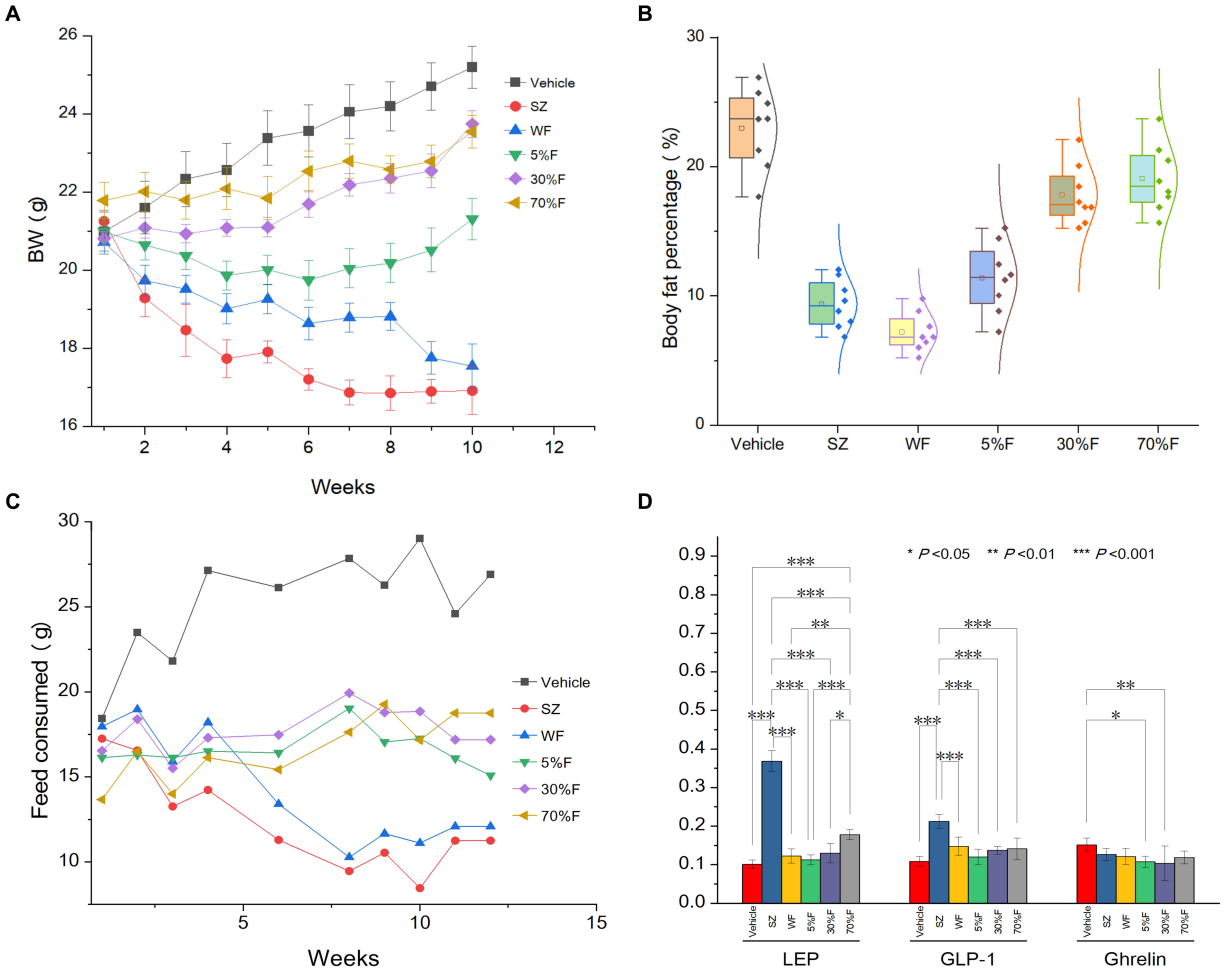
Influence of fractions on body weight, body fat, feed consumed, and LEP and GLP-1 of different treatment groups. **(A)** Body weight change for different groups (average ± standard deviation). **(B)** Body fat percentage of different groups (average ± standard deviation, normality). **(C)** Food intake amount change for different groups (average). **(D)** LEP, GLP-1, and Ghrelin levels in serum of different groups (average ± standard deviation, significance).

Through the weight, food intake, and body fat percentage data obtained from animal experiments, it was found that, compared with the vehicle group, the SZ group and the WF group significantly reduced weight (*p* < 0.01), food intake (*p* < 0.01), and body fat percentage (*p* < 0.01). The serum biochemical indicators showed that, after 10 weeks of oral administration of cedar needles, compared with the vehicle group, the mice in the experimental groups with LEP and GLP-1 showed significant increases, while the WF group showed no significant changes (Figure 1). Compared with the vehicle group, there were no significant changes in the blood serum levels of TC, TG, ATP, VHDL, HDL, and LDL in all experimental groups, suggesting that cedar pine needles may partially regulate weight and food intake in mice through leptin and GLP-1, but WF may not produce anti-obesity effects by regulating these targets. However, this is not the focus of this study, so it will not be further discussed. WF was selected for further component analysis based on the significance of weight and food intake changes.

### 3.3 Purification of active fraction

**Figure 2:**
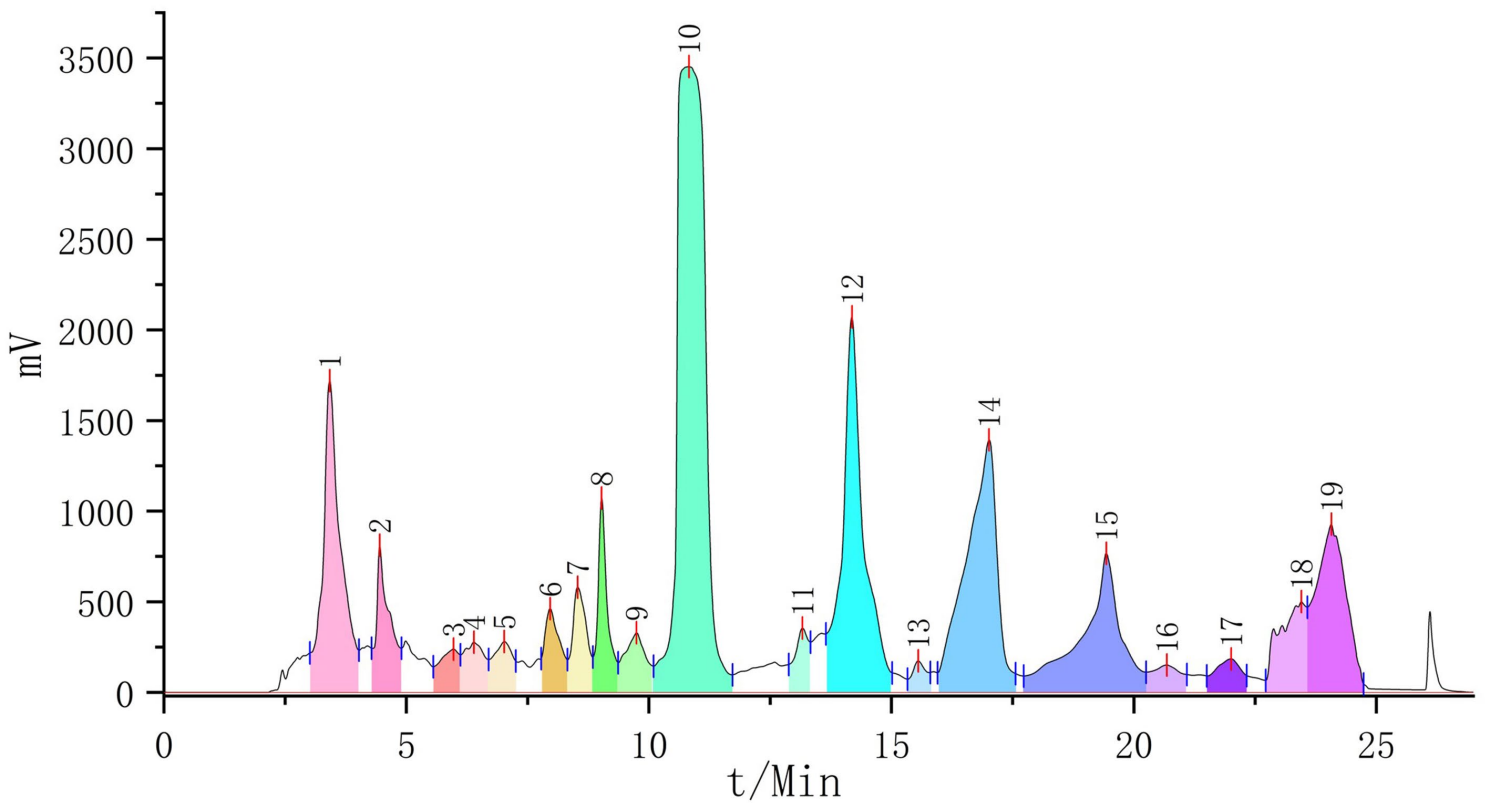
The preparation of liquid chromatography (19 fractions collected).

The active fraction was separated by preparative chromatography, yielding 19 fractions (Figure 2). After drying these fractions, it was found that their purity was insufficient for crystallization. Subsequently, glucose gel chromatography was used to further separate and purify these fractions, resulting in the identification of 19 potential monomeric compounds.

### 3.4 Characterization of isolated components

**Figure 3:**
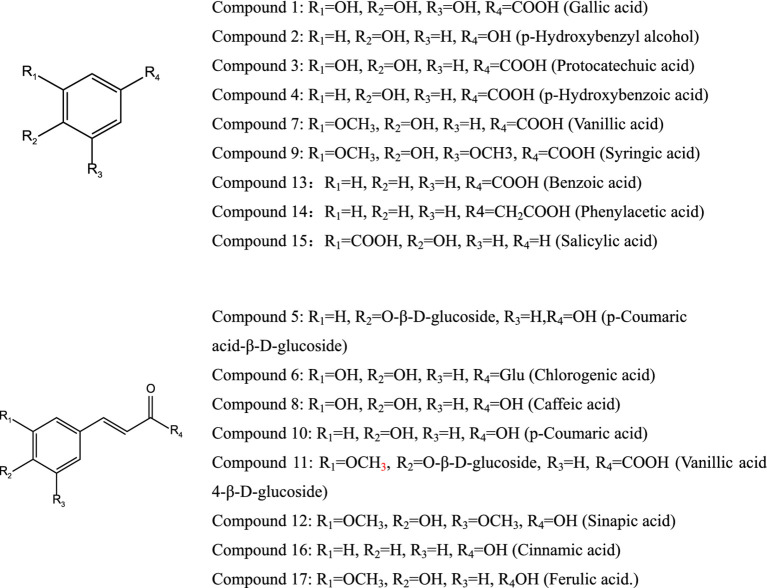
Chemical structure of monomer compounds.

A total of 19 potential monomeric compounds were obtained through experiments and analysis. Further purification confirmed the presence of 17 monomer compounds as shown in Figure 3: gallic acid (1), p-hydroxybenzyl alcohol (2), protocatechuic acid (3), p-hydroxybenzoic acid (4), p-coumaric acid-β-D-glucoside (5), chlorogenic acid (6), vanillic acid (7), caffeic acid (8), syringic acid (9), p-coumaric acid (10), vanillic acid-β-D-glucoside (11), sinapic acid (12), benzoic acid (13), phenylacetic acid (12), salicylic acid (15), cinnamic acid (16), and ferulic acid (17).

**Compound 1:** Gallic acid.

Appearance: White needle-shaped crystals.

^**1**^ **H-NMR (400 MHz, DMSO-d6):**

δ: 9.08 (2H, s, 3, 5-OH), 8.79 (1H, s, 4-OH), and 7.10 (2H, s, H-2, 6).

^**13**^**C-NMR (100 MHz, DMSO-d6):** δ: 119.2, (C-1), 108.3 (C-2, 6), 144.1 (C-3, 5), 137.3 (C-4), and 167.8 (C-7).

The NMR data are consistent with the literature report by Tian et al. (23). Therefore, the compound is identified as gallic acid.

**Compound 2:** p-hydroxybenzyl alcohol.

Appearance: White powder.

Molecular formula: C_7_H_8_O_2_.

^**1**^**H-NMR (400 MHz, DMSO-d6):** δ: 9.15 (s, 4-OH), 7.00 (2H, d, J = 8.1 Hz, H-2, 6), 6.66 (2H, d, J = 8.1 Hz, H-3, 5), and 4.43 (2H, m, 7-CH2).

^**13**^**C-NMR (100 MHz, DMSO-d6):** δ: 156.07 (C-4), 132.60 (C-1), 129.42 (C-3, 5), 115.67 (C-2, 6), and 63.56 (C-7).

The data are consistent with the literature report by Peng et al. (24). Therefore, compound 2 is identified as p-hydroxy benzyl alcohol.

**Compound 3:** Protocatechuic acid.

Appearance: White amorphous powder.

^**1**^**H NMR (400 MHz, CD**_**3**_**OD):** δH:7.31 (2H,m,H-2,6) and 6.68 (1H,d,J = 8.2 Hz,H-5).

^**13**^**C-NMR (100 MHz, CD**_**3**_**OD):** δC: 168.1(C-7), 149.3 (C-4), 144.2 (C-3), 121.1 (C-6), 120.9 (C-1), 115.3 (C-2), and 113.6 (C-5).

The data are consistent with the literature report (25). Therefore, compound 3 is identified as protocatechuic acid.

**Compound 4:** p-Hydroxybenzoic acid.

Appearance: White amorphous powder.

^**1**^**H-NMR (400 MHz, CD**_**3**_**OD):** δ 7.76 (2H, d, J = 8.0 Hz, H-2, 6) and 6.84 (2H, d, J = 8.0 Hz, H-3, 5).

^**13**^**C-NMR (100 MHz, CD**_**3**_**OD):** δ 120.4(C-1), 131.2 (C-2, 6), 113.8 (C-3, 5), 160.1(C-4), and 164.9 (C-1′).

The above NMR spectral data are consistent with the literature report by Tian et al. (23). Therefore, compound 4 is identified as p-hydroxybenzoic acid.

**Compound 5:** p-coumaric acid-β-D-glucoside.

Appearance: White powder.

Molecular formula: C_15_H_18_O_8_.

^**1**^**H-NMR (400 MHz, D**_**2**_**O):** δ: 7.46 (2H, d, J = 8.0 Hz, H-2,6), 7.03 (2H, d, J = 8.0 Hz, H-3,5), 6.33 (1H, d, J = 16.0 Hz, H-8), 7.26 (1H, d, J = 16.0 Hz, H-7), 5.05 (1H, d, J = 8.0 Hz, H-1′), and 3.42–3.88 (6H, m).

^**13**^**C-NMR (100 MHz, D2O):** δ: 176.20 (C-9), 122.77 (C-8), 140.30 (C-7), 130.09 (C-1), 129.35 (C-2,6), 157.44 (C-4), 116.68 (C-3,5), 99.83 (C-1′), 60.50 (C-6′), 69.40 (C-4′), 72.89 (C-2′), 75.51 (C-3′), and 76.12 (C-5′).

The above data are consistent with the reported findings by Peng et al. (24). Therefore, compound 5 is identified as p-coumaric acid-β-D-glucoside.

**Compound 6:** Chlorogenic acid.

Appearance: White powder.

^**1**^**H-NMR (400 MHz, CD**_**3**_**OD):** δ 7.05 (1H, s, H-2′), 6.93 (1H, d, J = 7.6, H-6′), 6.75 (1H, d, J = 7.6 Hz, H-5′), δ 7.55 (1H,d, J = 15.6 Hz, H-7′), 6.26 (1H, d, J = 15.6 Hz, H-8′), δ 5.35 (1H, m, H-3), 3.72(1H, s, H-4), 4.22(1H,d, J = 3.6 Hz, H-5), and δ 2.11–1.80 (4H, m, H-2, 6).

^**13**^**C-NMR (100 MHz, CD**_**3**_**OD):** δ 178.1 (C-7), 167.6(C-9′), δ: 149.2 (C-4′), 146.6 (C-3′), 127.5 (C-1′), 75.9 (C-1), δ: 146.6 (C-7′), 123.1 (C-6′), 116.5 (C-5′), 115.3 (C-8′), 115.6 (C-2′), 74.6 (C-4), 72.9 (C-3), 69.0 (C-5), 41.1 (C-6), and 36.5 (C-2).

The above data are consistent with the reported findings by Yao et al. (26). Therefore, compound 6 is identified as chlorogenic acid.

**Compound 7:** Vanillic acid.

Appearance: White powder.

Molecular formula: C_8_H_8_O_4_.

^**1**^**H-NMR (400 MHz, D**_**2**_**O):** δ7.37 ~ 7.41 (2H,m,H-2,6), 6.84 (1H,d,J = 8.0 Hz,H-5), 3.83 (OCH_3_).

^**13**^**C-NMR (100 MHz, D**_**2**_**O):** δ176.40 (C-7), 148.73 (C-3), 146.40 (C-4), 123.28 (C-1), 121.61 (C-6), 115.80 (C-2), 111.32 (C-5), and 55.88 (C-OCH_3_).

The above data are consistent with the reported findings by Peng et al. (24). Therefore, compound 7 is identified as vanillic acid.

**Compound 8:** Caffeic acid.

Appearance: White amorphous powder.

^**1**^**H-NMR (400 MHz, CD**_**3**_**OD):** δ 7.01 (1H, d, J = 2.4 Hz, H-2), 6.92 (1H, dd, J = 8.4, 2.4 Hz, H-6), 6.83 (1H, d, J = 8.4 Hz, H-5), 7.49 (1H, d, J = 15.6 Hz, H-7), and 6.21 (1H, d, J = 15.6 Hz, H-8).

The above data are consistent with the reported findings by Tian et al. (23). Therefore, compound 8 is identified as caffeic acid.

**Compound 9:** Syringic acid.

Appearance: White powder.

Molecular formula: C_9_H_10_O_5_.

^**1**^**H-NMR (400 MHz, CD**_**3**_**OD):** δ 7.25 (2H, s, H-2, 6) and 3.81 (3H, s, 3, 5-OCH_3_).

^**13**^**C-NMR (100 MHz, CD**_**3**_**OD):** δ 168.2 (COOH), 147.3 (C-3, 5), 140.2 (C-4), 121.1 (C-1), 106.9 (C-2, 6), and 54.9 (3, 5-OCH_3_).

The data are consistent with the literature reports by Long et al. (27). Therefore, compound 9 is identified as syringic acid.

**Compound 10:** p-coumaric acid.

Appearance: White powder.

Molecular formula: C_9_H_8_O_5_.

^**1**^**H-NMR (400 MHz, D**_**2**_**O):** δ 7.43 (1H, d, J = 16.0 Hz, H-7), 7.23 (2H, d, J = 8.0 Hz, H-2,6), 6.82 (2H, d, J = 8.0 Hz, H-3,5), and 6.28 (1H, d, J = 16.0 Hz, H-8).

^**13**^**C-NMR (100 MHz, D**_**2**_**O):** δ 176.20 (C-9), 157.60 (C-4), 140.79 (C-7), 129.57 (C-2,6), 127.21 (C-1), 115.95 (C-3,5), and 121.26 (C-8).

The data are consistent with the literature reports by Peng et al. (24). Therefore, compound 10 is identified as p-coumaric acid.

**Compound 11:** Vanillic acid-β-D-glucoside.

Appearance: White powder.

Molecular formula: C_14_H_16_O_9_.

^**1**^**H-NMR (400 MHz, D**_**2**_**O):** δ 7.08–7.21 (2H, m, H-2,6), 6.38 (1H, d, J = 8.0 Hz, H-5), 5.07 (1H, d, J = 8.0 Hz, H-1′), 3.43–3.84 (6H, m), and 3.83 (OCH3).

^**13**^**C-NMR (100 MHz, D2O):** δ 176.40 (C-7), 148.73 (C-3), 146.40 (C-4), 123.28 (C-1), 121.61 (C-6), 115.80 (C-2), 111.32 (C-5), 100.13 (C-1′), 60.40 (C-6′), 69.26 (C-4′), 72.79 (C-2′), 75.47 (C-3′), 76.11 (C-5′), and 55.88 (C-OCH_3_).

These data are consistent with the reported findings by Peng et al. (24). Therefore, compound 11 is identified as vanillic acid-β-D-glucoside..

**Compound 12:** Sinapic acid.

Appearance: Pale yellow powder.

^**1**^**H-NMR (400 MHz, DMSO-d6):** δ 7.42 (1H, d, J = 15.6 Hz, H-7), 6.82 (2H, s, H-2,6), 6.34 (1H, d, J = 12.8 Hz, H-8), and 3.79 (6H, s, 3,5-OMe).

^**13**^**C-NMR (100 MHz, DMSO-d6):** δ 167.6 (C-9), 147.1 (C-3,5), 144.3 (C-7), 137.1 (C-4), 124.5 (C-1), 115.2 (C-8), 105.5 (C-2,6), and 55.1 (3,5-OMe).

The data are consistent with the reported literature by Zhang et al. (28). Therefore, compound 12 is identified as sinapic acid.

**Compound 13:** Benzoic acid.

Appearance: White powder.

^**1**^**H-NMR (400 MHz, D**_**2**_**O):** δ 7.78 (d, 2H) and 7.40 (m,3H).

^**13**^**C-NMR (100 MHz,D**_**2**_**O):** δ 175.80 (C-7), 136.11 (C-4), 131.24 (C-1), 128.76 (C-C-2,6), and 128.27 (C-3,5).

These data are consistent with the reported findings by Peng et al. (24). Therefore, compound 13 is identified as benzoic acid.

**Compound 14:** Phenylacetic acid.

Appearance: White wax-like substance.

^**1**^**H-NMR (400 MHz,CD**_**3**_**OD):** δ 7.16 ~ 7.28 (5H, m, H-2 ~ 6), 3.49 (2H, s, H-7).

^**13**^**CNMR (100 MHz, CD**_**3**_**OD):** δ 134.2 (C-1), 128.1 (C-2,6), 126.8 (C-3,5), 125.4 (C-4), 40.1 (C-7), and 174.8 (C-8).

The data are consistent with the literature reports by Si et al. (29). Therefore, compound 14 is identified as phenylacetic acid.

**Compound 15:** Salicylic acid.

Appearance: White powder.

^**1**^**H-NMR (400 MHz, CD**_**3**_**OD):** δ 7.81 (1H, d, J = 8.4 Hz, H-6), 7.39 (1H, m, H-4), 6.85 (1H, d, J = 8.0 Hz, H-5), and 6.79 (1H, m, H-3).

These data are consistent with the reported literature by C. MA et al. (30). Therefore, compound 15 is identified as salicylic acid (acetylsalicylic acid).

**Compound 16:** Cinnamic acid.

Appearance: A white, crystalline substance.

^**1**^**H-NMR (400 MHz, CD3OD):** δ 7.55 (1H, d, J = 16.0 Hz, H-7), 6.32 (1H, d, J = 16.0 Hz, H-8), 7.65 (2H, m), and 6.87 (3H, m).

^**13**^**C-NMR (100 MHz, CD3OD):** δ 168.35 (C-9), 145.15 (C-7), 132.16 (C-4), 129.72 (C-2,6), 129.02 (C-8), 125.76 (C-1), and 115.43 (C-3, 5).

The data are consistent with the reported findings in the literature by D. Peng et al. (24). Therefore, compound 16 is identified as cinnamic acid.

**Compound 17:** Ferulic acid.

Appearance: White needle crystals.

^**1**^**H-NMR (400 MHz, DMSO-d6):** δ 7.92 (1H, d, J = 15.8 Hz, H-7), 7.36 (1H, d, J = 2.4 Hz, H-2), 7.18 (1H, d, J = 5.4 Hz, H-6), 7.09 (1H, d, J = 8.4 Hz, H-5), 6.72 (1H, d, J = 15.8 Hz, H-8), and 3.66 (3H, s, -OCH_3_).

^**13**^**C-NMR (100 MHz, DMSO-d6):** δ 130.6 (C-1), 120.3 (C-2), 152.1 (C-3), 154.6 (C-4), 120.8 (C-5), 125.8 (C-6), 147.4 (C-7), 116.1 (C-8), 172.3 (C-9), and 56.8 (-OCH_3_).

These data are consistent with the reported findings by Zhang et al. (31). Therefore, compound 17 is identified as ferulic acid.

### 3.5 Determination

**Figure 4:**
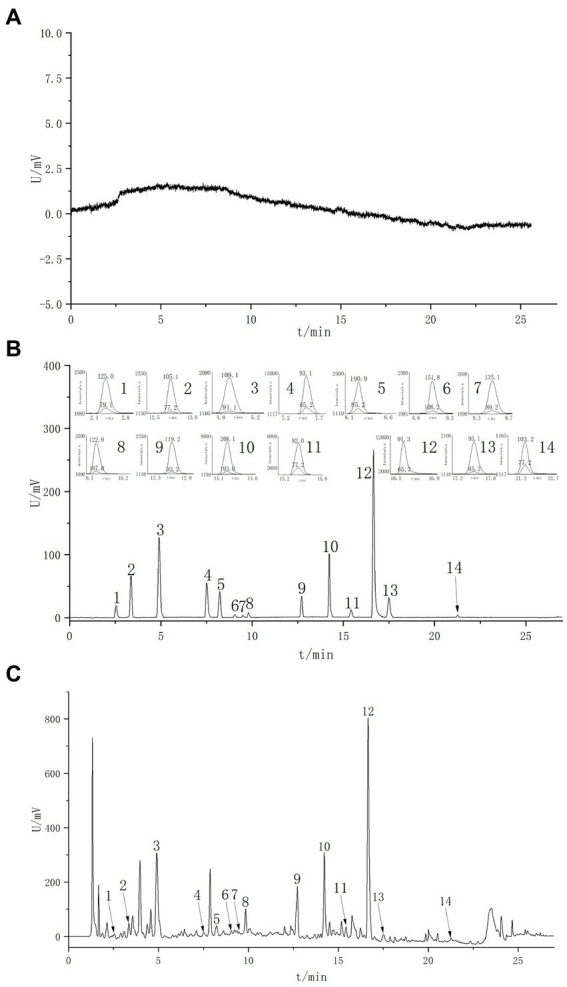
Extracted ion current chromatograms of various constituents in MRM mode. **(A)** Blank spectrum. **(B)** Standard spectrum. **(C)** Sample spectrum. 1. Gallic acid; 2. P-Hydroxybenzyl alcohol; 3. Protocatechuic acid; 4. P-Hydroxybenzoic acid; 5. Chlorogenic acid; 6. Vanillic acid; 7. Caffeic acid; 8. Syringic acid; 9. P-Coumaric acid; 10. Sinapic acid; 11. Benzoic acid; 12. Phenylacetic acid; 13. Sallcylic acid; 14. Trans-Cinnamic acid.

The content of phenolic acids in cedar pine needles was determined using an external standard method. The total contents of 14 phenolic acids were found to be 55.35 mg·g-1 (S1) and 46.22 mg·g-1 (S2). Among the 14 phenolic acids, benzene acetic acid had the highest concentration in both samples (Table 2, Figure 4).

**Table 2:** Results of determination of different kinds of pine needles (mg·g^−1^).

	Contents (mg·g^−1^)													
	1	2	3	4	5	6	7	8	9	10	11	12	13	14
S1	1.33	5.21	5.15	3.02	0.87	2.12	0.58	1.20	2.14	8.69	3.77	18.89	2.25	0.13
S2	1.08	3.34	4.88	0.15	4.29	0.73	0.22	0.39	1.54	11.25	2.08	15.37	0.89	0.01

### 3.6 Method validation

#### 3.6.1 Specificity verification

A 50% methanol solution was used as a blank sample for analysis. The results showed that the blank sample did not produce any response within the retention time range of the control product, indicating no interference with the determination of the tested component (Figure 4).

#### 3.6.2 LOQ and LOD

The results showed that the signal-to-noise ratio of the substances measured in the content determination and methodological verification processes were all greater than 1:10. Under the conditions specified in “2.11 MS method development,” based on a sample injection volume of 2 μL, the limits of quantification for gallic acid, p-hydroxybenzyl alcohol, protocatechuic acid, p-hydroxybenzoic acid, chlorogenic acid, vanillic acid, caffeic acid, syringic acid, p-coumaric acid, sinapic acid, benzoic acid, phenylacetic acid, salicylic acid, and trans-cinnamic acid were approximately 0.1 ng, 0.2 ng, 0.03 ng, 0.2 ng, 0.05 ng, 0.1 ng, 0.4 ng, 0.3 ng, 0.1 ng, 0.3 ng, 0.2 ng, 0.2 ng, 0.06 ng, and 0.01 ng, respectively. The LOD was 0.3 times the LOQ for each acid mentioned above.

#### 3.6.3 Repeatability experiment

The relative standard deviations (RSDs) for gallic acid, p-hydroxybenzyl alcohol, protocatechuic acid, p-hydroxybenzoic acid, chlorogenic acid, vanillic acid, caffeic acid, syringic acid, p-coumaric acid, sinapic acid, benzoic acid, phenylacetic acid, salicylic acid, and trans-cinnamic acid were 0.58, 2.01, 1.32, 0.25, 2.08, 1.47, 1.62, 0.95, 1.38, 1.52, 2.12, 0.59, 1.77, and 1.45%, respectively..

#### 3.6.4 Precision

We took the prepared mixed standard solution mentioned in Section 2.8 and then proceeded to determine its composition six times continuously. The RSDs of the response area under the curve (AUC) for the 14 components were 0.79, 1.64, 2.06, 1.48, 1.15, 2.33, 1.29, 0.43, 1.26, 0.87, 1.04, 1.51, and 2.46%.

#### 3.6.5 Linearity and range

Linearity was evaluated within the concentration range of 100 times the initial concentration ranges. The calibration curves showed good correlation coefficients (R^2^ > 0.999) over the calibration range selected for the target compounds (Table 3).

**Table 3:** Linear range, regression equation, and the correlation coefficient of 14 components.

Components	Range (ng)	Equation of linearity	Correlation coefficient
Gallic acid	84.80 ~ 0.85	*y* = 5,866*x* + 10.3096	*R*^2^ = 0.9997
p-Hydroxybenzyl alcohol	214.80 ~ 2.15	*y* = 73,435*x* − 32.999	*R*^2^ = 0.9998
Protocatechuic acid	120.64 ~ 1.21	*y* = 19,782*x* + 1.5104	*R*^2^ = 0.9996
p-Hydroxybenzoic acid	459.20 ~ 4.59	*y* = 38,329*x* − 249.23	*R*^2^ = 0.9995
Chlorogenic acid	424.0 ~ 4.24	*y* = 139,250*x* − 211.82	*R*^2^ = 0.9998
Vanillic acid	137.20 ~ 1.37	*y* = 41,022*x* − 205.815	*R*^2^ = 0.9998
Caffeic acid	139.48 ~ 1.39	*y* = 45,265*x* − 388.24	*R*^2^ = 0.9995
Syringic acid	126.40 ~ 1.26	*y* = 24,056*x* + 34.514	*R*^2^ = 0.9996
p-Coumaric acid	212.16 ~ 2.12	*y* = 54,634*x* − 264.51	*R*^2^ = 0.9994
Sinapic acid	446.0 ~ 4.46	*y* = 158,448*x* − 1001.9	*R*^2^ = 0.9996
Benzoic acid	404.8 ~ 4.05	*y* = 120,417*x* + 476.7	*R*^2^ = 0.9999
Phenylacetic acid	435.84 ~ 4.36	*y* = 118,439*x* + 69.867	*R*^2^ = 0.9998
Salicylic acid	75.20 ~ 0.75	*y* = 25,161*x* − 118.4	*R*^2^ = 0.9996
Cinnamic acid	7.07 ~ 0.07	*y* = 4205.3*x* + 17.022	*R*^2^ = 0.9995

#### 3.6.6 Accuracy

Two injections were processed for each sample, and the average recovery and RSDs were calculated using SPSS software (Table 4).

**Table 4:** Determination results of the recovery rate.

	Content (mg)	Added (mg)	Found (mg)	Recovery (%)	RSDs (%, *n* = 3)
Gallic acid	0.266	0.2072	0.465	96.04%	3.45%
	0.266	0.259	0.531	102.32%	
	0.266	0.3108	0.582	101.67%	
p-Hydroxybenzyl alcohol	1.042	0.84	1.834	94.29%	2.68%
	1.042	1.05	2.054	96.38%	
	1.042	1.26	2.295	99.44%	
Protocatechuic acid	1.03	0.82	1.839	98.66%	3.26%
	1.03	1.025	2.108	105.17%	
	1.03	1.23	2.271	100.89%	
p-Hydroxybenzoic acid	0.604	0.4688	1.085	102.60%	3.40%
	0.604	0.586	1.167	96.08%	
	0.604	0.7032	1.314	100.97%	
Chlorogenic acid	0.174	0.1448	0.320	100.83%	3.26%
	0.174	0.181	0.349	96.69%	
	0.174	0.2172	0.398	103.13%	
Vanillic acid	0.424	0.348	0.768	98.85%	0.94%
	0.424	0.435	0.846	97.01%	
	0.424	0.522	0.935	97.89%	
Caffeic acid	0.116	0.0968	0.215	102.27%	3.21%
	0.116	0.121	0.242	104.13%	
	0.116	0.1452	0.258	97.80%	
Syringic acid	0.24	0.188	0.426	98.94%	2.21%
	0.24	0.235	0.483	103.40%	
	0.24	0.282	0.525	101.06%	
p-Coumaricacid	0.428	0.332	0.752	97.59%	2.22%
	0.428	0.415	0.837	98.55%	
	0.428	0.498	0.935	101.81%	
Sinapic acid	1.738	1.3984	3.157	101.47%	3.34%
	1.738	1.748	3.414	95.88%	
	1.738	2.0976	3.747	95.78%	
Benzoicacid	0.754	0.604	1.341	97.19%	0.49%
	0.754	0.755	1.495	98.15%	
	0.754	0.906	1.638	97.57%	
Phenylacetic acid	3.778	3.1352	6.878	98.88%	1.94%
	3.778	3.919	7.549	96.22%	
	3.778	4.7028	8.477	99.92%	
Salicylic acid	0.45	0.3408	0.795	101.23%	2.86%
	0.45	0.426	0.893	103.99%	
	0.45	0.5112	0.952	98.20%	
Cinnamic acid	0.026	0.0224	0.048	98.21%	2.44%
	0.026	0.028	0.053	96.43%	
	0.012	0.014	0.026	97.35%	

## 4 Conclusion

Research on phenolic acids in pine needles is relatively limited due to two main reasons. First, our study found that polysaccharides in pine needles have highly similar polarity and solubility to phenolic acids, making it difficult to separate these substances of similar molecular weight and polarity using common chromatographic separation methods such as silica gel columns, macroporous resin columns, polyacrylamide columns, and glucose gel columns. Second, small molecular-weight phenolic acids due to their simple structures are widely present in plants but often do not attract much attention.

For a long time, phenolic compounds have been the subject of extensive study. In addition to their traditional antioxidant and antibacterial activities, phenolic compounds have also been found to regulate α-amylase activity, improve insulin resistance, enhance gut microbiota, and resist obesity. These compounds even possess potential as therapy against snakebites. Pine needles are rich in natural antioxidants, and the phenolic acid content is related to their total antioxidant capacity. Therefore, phenolic acids can be an important dietary source in pine needles to prevent diseases caused by oxidative stress.

In addition, the carboxyl groups in smaller-molecular phenolic acids can be combined with lipids in the bacterial cell membrane to form carboxylate sodium salts, reducing the hydrophobicity of the cell membrane surface, thereby affecting the permeability of the bacterial cell wall and the absorption of amino acids, forming the main basis of the antibacterial activity of plants. Moreover, phenolic acids can affect the activities of α-glucosidase and α-amylase, slowing down the decomposition and absorption rates of carbohydrates. Currently, it is not clear whether phenolic acids affect energy uptake and metabolism by influencing digestive enzymes and the environment of the gastrointestinal microbiomes.

Therefore, it is necessary to conduct in-depth research on the metabolism and bioavailability of phenolic acids in edible plants. Although the mechanism of the activity needs to be further studied, it is clear that WF, which is rich in phenolic compounds, has potential for further development and research.

Subsequent studies found that the 14 components determined in this study are commonly found in various pine needles (needle leaves of *Cedrus deodara* (Roxb.) G. Don., *Pinus bungeana* Zucc. ex Endl., *Pinus tabuliformis* Carriere, *Pinus sylvestris* var. mongholica Litv., *Pinus armandii* Franch., and *Larix gmelinii* (Rupr.) Kuzen.). Among them, nine substances, including p-hydroxybenzoic acid, chlorogenic acid, vanillic acid, syringic acid, p-coumaric acid, sinapic acid, benzoic acid, and salicylic acid, were isolated, identified, and determined for the first time in pine needles. These components are mainly phenolic acids, which may contribute to the antioxidant and antibacterial activities of pine needles. They may also be the main source of allelopathic effects and gastrointestinal tract stimulation. Therefore, this study holds significant reference value for the precise and scientific application of pine needles in the future.

## Data availability statement

The original contributions presented in the study are included in the article/supplementary material, further inquiries can be directed to the corresponding authors.

## Ethics statement

The animal study was approved by Animal Ethics Committee of Lanzhou food and drug testing institute. The study was conducted in accordance with the local legislation and institutional requirements.

## Author contributions

XW: Conceptualization, Data curation, Formal analysis, Investigation, Methodology, Writing – original draft. BL: Data curation, Formal analysis, Investigation, Software, Validation, Writing – original draft. DL: Resources, Writing – original draft. YG: Data curation, Writing – original draft. JZ: Methodology, Writing – original draft. WL: Methodology, Writing – original draft. TP: Validation, Writing – original draft. QM: Project administration, Writing – review & editing. XS: Funding acquisition, Supervision, Writing – review & editing.
